# Prognostic Role of NLR, PLR and MHR in Patients With Idiopathic Pulmonary Fibrosis

**DOI:** 10.3389/fimmu.2022.882217

**Published:** 2022-04-28

**Authors:** Yiran Chen, Jingya Cai, Mengmeng Zhang, Xin Yan

**Affiliations:** Department of Respiratory and Critical Care Medicine, Nanjing Drum Tower Hospital, The Affiliated Hospital of Nanjing University Medicine School, Nanjing, China

**Keywords:** neutrophil-lymphocyte ratio, platelet-lymphocyte ratio, monocyte-high density lipoprotein ratio, idiopathic pulmonary fibrosis, overall survival

## Abstract

**Background:**

Idiopathic pulmonary fibrosis (IPF) is a chronic, progressive interstitial lung disease with low survival time. Since the pathophysiological progression of IPF is closely associated with immunological and inflammatory responses, immune biomarkers, including neutrophil-lymphocyte ratio (NLR), platelet-lymphocyte ratio (PLR), and monocyte-high density lipoprotein ratio (MHR), have the potential to predict overall survival in IPF patients.

**Methods:**

A total of 278 patients with IPF were finally enrolled. The demographic and clinical characteristics of the patients at baseline were recorded. Multivariable Cox regression analysis was used to evaluate the association between the three biomarkers and overall survival in both the total cohort and acute exacerbation subgroup.

**Results:**

The median follow-up was 5.84 months. After adjusting for confounders, we found that only elevated NLR was associated with worse overall survival (OR = 1.019, 95% CI 1.001-1.037, *P* =0.041) by using multivariable Cox regression analysis. In 116 acute exacerbation IPF patients, the results of the Cox multiple regression model also indicated that the NLR was a significant prognostic factor (OR= 1.022, 95% CI 1.001-1.044, *P* =0.036). The NLR before death was also significantly higher than that at admission in nonsurvival acute exacerbation IPF patients (*P*=0.014). No significant differences were found in PLR (P=0.739) or MHR changes (P=0.478).

**Conclusions:**

Our results indicated that elevated NLR expression is associated with shorter overall survival in IPF patients, which is independent of other prognostic factors. The NLR may be regarded as a reliable prognostic biomarker for IPF patients.

## Introduction

Idiopathic pulmonary fibrosis (IPF) is a chronic, progressive, fibrotic interstitial lung disease of unknown causes with high mortality (1, 2). The mean life expectancy is only 2-3 years in the absence of lung transplantation (2). The majority of patients usually experience a gradual worsening of exertional dyspnea and reductions in lung function (1). Acute exacerbation (AE) may occur in a minority proportion of IPF patients, with a significantly high in-hospital mortality rate of more than 50% despite active treatment (3, 4). Currently, only two medicines, nintedanib and pirfenidone, are approved by the Food and Drug Administration for the treatment of IPF (5). Because the disease course of IPF is highly variable, the prognosis of IPF is difficult to predict (6), which is raising an urgent need for identifying reliable biological markers, especially noninvasive serum markers, in evaluating the severity of the disease, predicting disease progression and therapeutic effects on treatment, and measuring the treatment response (7).

The pathogenesis of IPF is a complex genetic disorder. The formation of IPF is gradually converted from a single chronic inflammatory disorder to a comprehensive consequence of epithelial cell injury, chronic inflammation, fibroblast proliferation, and deposition of extracellular matrix (8, 9). Inflammation and oxidative stress further lead to epithelial cell injury and fibroblast activation and migration in aging alveolar epithelium as local microinjuries (2).

The neutrophil lymphocyte ratio (NLR), platelet–lymphocyte ratio (PLR) and monocyte-high density lipoprotein ratio (MHR) are biomarkers of inflammation and oxidative stress. The expression of NLR and PLR is associated with worse outcomes in multiple diseases, including chronic obstructive pulmonary disease, coronavirus-induced disease 2019 (COVID-2019), pulmonary embolism, cardiovascular diseases, rheumatoid arthritis and various solid tumors (10–12). The MHR has also been regarded as a prognostic marker in cardiovascular diseases (13, 14).

However, limited data have been presented on the relationship between NLR/PLR/MHR and clinical outcomes in IPF patients. There are a few studies evaluating the prognostic value of NLR and/or PLR in IPF patients, suggesting that higher NLR and/or PLR may contribute to worse outcomes (15–17). Although high lipid protein levels have been found to have a negative correlation with the mortality rate in IPF patients, it is still unknown whether the MHR is a prognostic biomarker for evaluating the progression of IPF patients (18). Additionally, the number of monocytes is an independent risk factor for IPF progression, all-cause hospitalization and all-cause mortality (19).

Consequently, the aim of this retrospective study was to determine the prognostic value between NLR/PLR/MHR and overall survival in patients with IPF. We also investigated the changes in NLR/PLR/MHR at baseline and before death in AE-IPF patients.

## Materials and Methods

### Patients and Data Collection

A retrospective review of the medical records of 613 patients diagnosed with idiopathic pulmonary fibrosis at Drum Tower Hospital from June 2017 to June 2021 was carried out. All patients were over 18 years old and had clinical lymphocyte, neutrophil, and monocyte counts and a high density of lipoprotein within 24 h after admission. The exclusion criteria were as follows: 1) incomplete data; 2) severe, life-threatening disease of another system unrelated to idiopathic pulmonary fibrosis; and 3) confirmed lung cancer before admission. A total of 335 patients were excluded based on these criteria. Finally, 278 patients were enrolled in our final analysis.

The diagnosis of idiopathic pulmonary fibrosis was performed according to the 2018 international consensus guidelines (20), which depends on the identification of the usually interstitial pneumonia (UIP) pattern, usually with high-resolution computed tomography (HRCT), and rules out other interstitial lung diseases or overlapping conditions. The diagnostic criteria for AE-IPF were based on the 2018 guidelines (20), which include a previous or concurrent diagnosis of IPF and meeting the following criteria: acute worsening or development of dyspnea, typically<1 month (3); the appearance of new bilateral ground-glass opacity and/or consolidation superimposed consistent with UIP in HRCT imaging; and (4) deterioration not fully explained by cardiac failure or fluid overload.

We collected the following variables for all patients at baseline: demographic and laboratory characteristics. The follow-up time was defined as the interval from admission to death, the last visit or the end of the study. Follow-up data were obtained by inpatient/outpatient visits or telephone calls every year. We also collected repeated measurements of NLR/PLR/MHR in non-survival AE-IPF patients on the last hospital admission and before death. All methods were carried out in accordance with relevant guidelines and regulations. PaO2 was measured with arterial blood sampling and FiO2=(21+oxygen flow*4)/100.This study was approved by the Human Research Ethics Committee of Drum Tower Hospital. The informed consent requirement was exempted because of the retrospective study.

### Statistical Analyses

Quantitative variables with a normal distribution are expressed as the mean and standard deviation, and the difference was identified by using Student’s t test. Variables that were not normally distributed are presented as the median and range, and the Mann–Whitney–Wilcoxon test was used for comparisons. Categorical variables were described as numbers and frequencies and compared using Pearson’s chi-square or Fisher’s exact tests.

First, we analyzed the correlation between the baseline NLR/PLR/MHR and PaO2/FiO2 in the total cohort. Then, we analyzed the relationship between the baseline NLR and survival in the total cohort and AE-IPF subgroup separately. In the total cohort, we also divided the patients into two groups according to the median of the baseline NLR/PLR/MHR values. We used multivariable Cox regression analysis and smooth curve fitting to test the independent effects of the baseline NLR/PLR/MHR and mortality with unadjusted and adjusted models. Then, we selected statistically significant variables between survivors and nonsurvivors at baseline as confounders to adjust the regression model. In the AE-IPF subgroup, univariate analysis was used to assess the potential influence of patient characteristics on prognosis. Statistically significant factors associated with NLR/PLR/MHR were introduced into a multivariate Cox proportional hazards analysis. In addition, we compared the changes in NLR/PLR/MHR in AE-IPF patients who experienced in-hospital mortality using the Mann–Whitney–Wilcoxon test. The area under the receiver operating characteristic (AUROC) curve was created to estimate the predictive power of NLR/PLR/MHR for overall survival.

All missing data were imputed using the E-M model. All tests were two-sided, and a *P* value < 0.05 was defined as statistically significant. R software, version 3.5.1 (http://www.r-project.org), was used for all analyses.

## Results

### Baseline Characteristics of the Study Population

The baseline characteristics of 278 IPF patients are summarized in **Table 1**, which contained 117 survivors and 161 non-survivors. The mean age of the study cohort was 67.97 ± 8.93 years, and 79.14% of the participants were male. The median NLR values at baseline were 4.49 ± 4.76 in survivors and 8.18 ± 11.60 in nonsurvivors. The median PLR values at baseline were 150.22 ± 91.84 in survivors and 202.80 ± 218.76 in nonsurvivors. The median MHR values at baseline were 0.51 ± 0.36 in survivors and 0.53 ± 0.45 in nonsurvivors. The demographic characteristics at baseline were generally balanced between the two groups. Non-survivors had higher proportion of AE-IPF patients and lower PaO2/FiO2 than survivors. We also found that there were significant differences in laboratory data. Non-survivors had markedly higher counts of white blood cells (WBCs) and neutrophils and higher levels of lactate dehydrogenase (LDH), total cholesterol (TC), apolipoprotein B (apoB), arcinoembryonic antigen (CEA), cytokeratin 21-1 (CYFRA21-1) and neurospecific enolase (NSE) than survivors.

**Table 1:** Baseline characteristics of the total cohort.

Variables	Survivors (n=117)	Nonsurvivors (n=161)	*P*
Age (years)	67.62 ± 8.60	68.45 ± 9.39	0.37
Male (n,%)	128 (79.50)	92 (78.63)	0.86
Group			0.02
Stable (n,%)	103 (63.98)	59 (50.43)	
AE (n,%)	58 (36.02)	58 (49.57)	
Smoking (n,%)	68 (42.24)	40 (34.19)	0.17
Disease duration (months)	19.28 ± 15.61	8.06 ± 11.27	<0.01
PaO2/FiO2	329.19 ± 125.35	264.54 ± 122.64	<0.01
WBC count (10^9/L)	7.54 ± 3.11	9.07 ± 3.61	<0.01
Neutrophils (10^9/L)	5.20 ± 2.86	6.84 ± 3.53	<0.01
Lymphocytes (10^9/L)	1.66 ± 0.68	1.54 ± 0.83	0.10
Monocytes (10^9/L)	0.48 ± 0.25	0.49 ± 0.29	0.95
Platelet (10^9/L)	194.67 ± 81.35	210.02 ± 88.43	0.14
ALT (U/L)	21.11 ± 16.66	23.25 ± 16.02	0.08
AST (U/L)	20.07 ± 8.95	22.40 ± 11.55	0.16
LDH (U/L)	256.86 ± 73.73	344.09 ± 174.89	<0.01
ALP (U/L)	74.06 ± 21.69	76.08 ± 28.06	0.56
TB (umol/L)	10.19 ± 4.78	11.57 ± 5.81	0.07
TC (mmol/L)	4.12 ± 1.02	4.36 ± 0.99	0.04
HDL (mmol/L)	1.11 ± 0.34	1.11 ± 0.38	0.91
LDL (mmol/L)	2.41 ± 0.75	2.51 ± 0.78	0.26
ApoA (g/L)	0.98 ± 0.26	0.98 ± 0.29	0.88
ApoB (g/L)	0.76 ± 0.21	0.85 ± 0.25	<0.01
CRP (ng/L)	19.44 ± 30.35	27.55 ± 41.00	0.05
D-dimer (mg/L)	1.48 ± 5.54	3.84 ± 13.48	0.05
CEA (ng/ml)	3.52 ± 2.46	5.17 ± 4.16	0.001
CYFRA21-1 (ng/ml)	5.10 ± 2.90	7.16 ± 4.70	0.011
NSE (ng/ml)	15.24 ± 5.09	19.84 ± 9.42	<0.01
NLR	4.49 ± 4.76	8.18 ± 11.60	0.002
PLR	150.22 ± 91.84	202.80 ± 218.76	0.106
MHR	0.51 ± 0.36	0.53 ± 0.45	0.416

AE, acute exacerbation; WBC, white blood cell; ALT, alanine aminotransferase; AST, glutamic oxaloacetic transaminase; LDH, lactate dehydrogenase; ALP, alkaline phosphatase; TC, total cholesterol; TB, total biliburin; HDL, high density lipoprotein; LDL, low density lipoprotein; ApoA, Apolipoprotein A; ApoB, Apolipoprotein B; CRP, C-reactive protein; CEA, carcinoembryonic antigen; CYFRA21-1, cytokeratin 21-1; NSE, neurospecific enolase; NLR, neutrophil-lymphocyte ratio; PLR, platelet–lymphocyte ratio; MHR, monocyte-high density lipoprotein ratio.

### Association Between Baseline NLR/PLR/MHR and PaO2/FiO2

**Figure 1:**
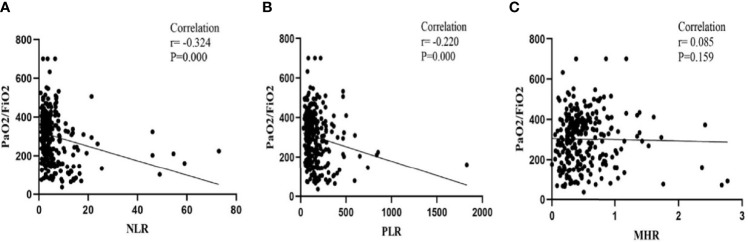
Correlations of the NLR, PLR and MHR with PaO2/FiO2 on admission: **(A)** Correlations of the NLR with PaO2/FiO2; **(B)** Correlations of the PLR with PaO2/FiO2; **(C)** Correlations of the MHR with PaO2/FiO2.

As shown in **Figure 1**, the relationship between NLR/PLR/MHR and PaO2/FiO2 was displayed. The expression of NLR and PLR was negatively associated with PaO2/FiO2 (NLR: r=-0.324, *P*<0.001; PLR: r=-0.220, *P*<0.001). However, no association was observed between MHR and PaO2/FiO2 (r=0.085, *P*=0.159).

### Association Between Baseline NLR/PLR/MHR and Overall Survival in All Patients

**Figure 2:**
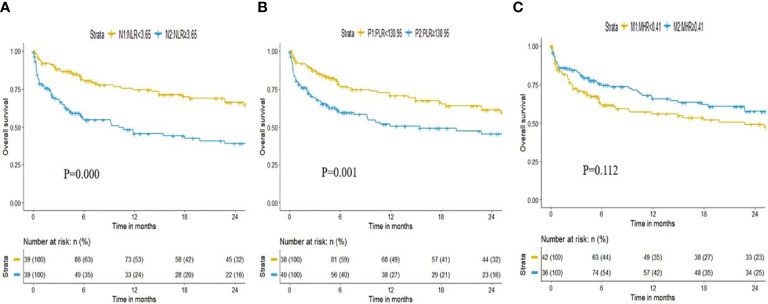
Kaplan–Meier curves for overall survival time by NLR, PLR and MHR: **(A)** Kaplan–Meier curves for overall survival time by NLR. **(B)** Kaplan–Meier curves for overall survival time by PLR; **(C)** Kaplan–Meier curves for overall survival time by MHR.

The median follow-up time of all patients was 5.84 (2.19–23.44) months. During the follow-up period, 117 patients died. We divided all included patients into 2 groups according to the median NLR/PLR/MHR value (NLR: 3.65, PLR 130.95, MHR 0.41), and the Kaplan–Meier survival curves are shown in **Figure 2**. Higher expression of NLR and PLR were associated with shorter overall survival time [N2: 3.93 (0.77-11.90) months vs N1: 14.10 (4.60-28.07) months, OR(95%CI): 2.114 (1.459-3.064), *P* = 0.000; P2: 4.52 (1.07-14.60) months vs P1: 11.92 (3.60-28.47) months, OR(95%CI):1.872(1.294-2.709), *P* = 0.001], while patients with the higher level of MHR showed a longer survival time [M2: 7.88 (2.93-24.34) months vs M1: 5.60 (1.60-19.83) months, OR(95%CI): 0.744(0.516-1.071), *P* = 0.112].

**Figure 3:**
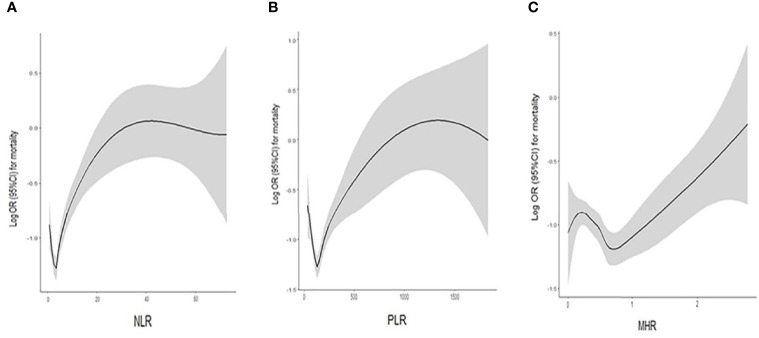
A smooth curve fitting for the relationship between the baseline NLR, PLR, MHR and the log odds ratio of mortality: **(A)** An inverse U-shaped relationship between NLR and mortality; **(B)** An inverse U-shaped relationship between PLR and mortality; **(C)** An U-shaped relationship between MHR and mortality.

**Figure 4:**
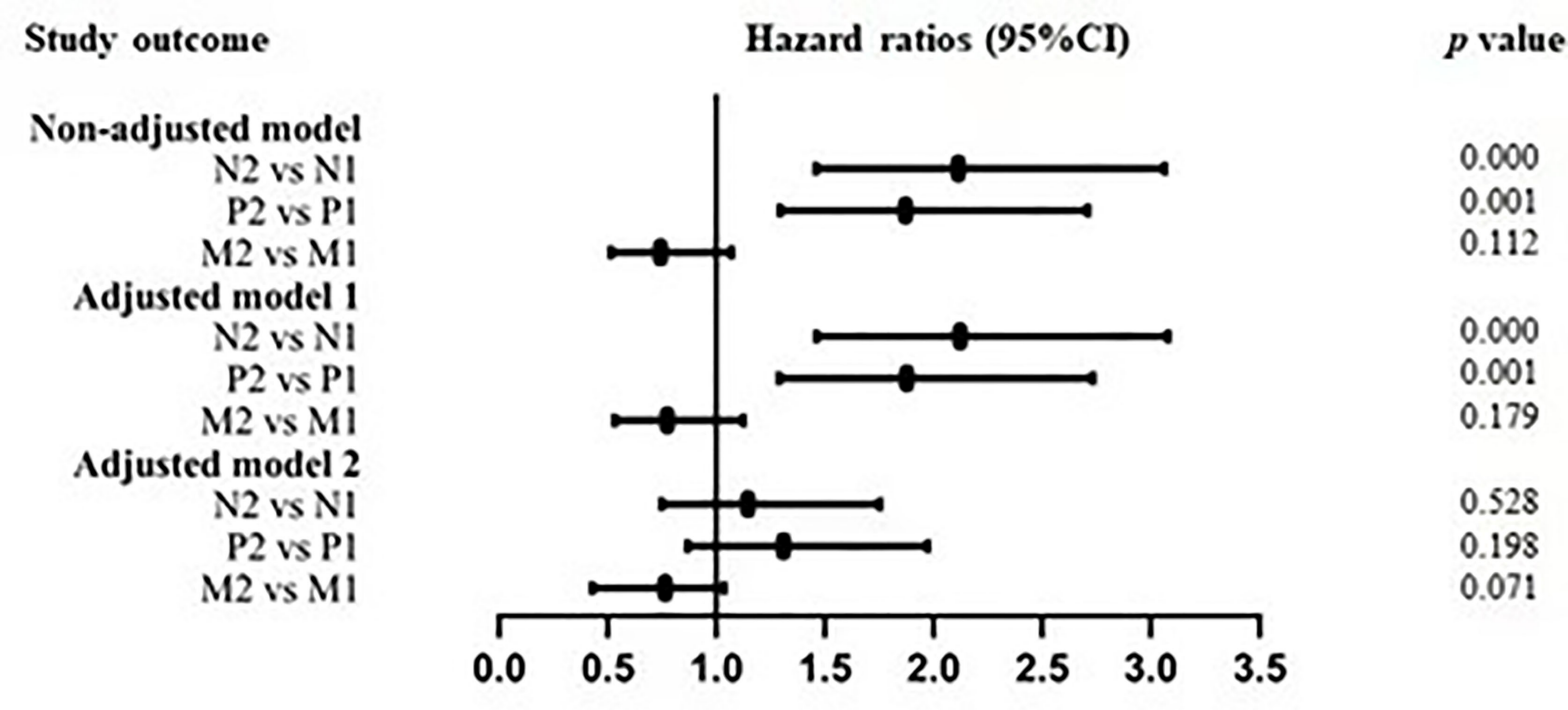
Adjusted hazard ratios for mortality by NLR, PLR and MHR.

As shown in **Table 2**, multivariable Cox regression analysis of baseline NLR, PLR and MHR for overall survival was performed. After adjusted for confounding factors including age, sex, smoking, PaO2/FiO2, WBCs, LDH, TC, apoB, CEA, CYFRA21-1 and NSE, an SD increase in the baseline NLR was associated with a 1.9% increase in the risk of mortality (OR 1.019, 95% CI 1.001-1.037, *P* =0.041). However, PLR and MHR were not independent risk factors for mortality after adjusted for confounding factors. Smooth curve fitting was used to indicate the correlation between the baseline NLR/PLR/HMR and the risk of mortality, which is presented in **Figure 3**. In the generalized regressive model, an inverse U-shaped relationship between NLR and mortality, an inverse U-shaped relationship between PLR and mortality and a U-shaped relationship between MHR and mortality were observed after adjusting for potential confounders. After the expression of NLR, PLR and MHR were classified into two categories, there was no significant association between NLR, PLR, MHR and mortality in adjusted model 2 (**Table 2** and **Figure 4**).

**Table 2:** Multivariable Cox regression analysis of baseline NLR, PLR and MHR for overall survival.

Variables	Non adjusted			Adjusted model 1			Adjusted model 2		
	*OR*	95%*CI*	*P*	*OR*	95%*CI*	*P*	*OR*	95%*CI*	*P*
NLR (1sd)	1.040	1.023-1.050	<0.001	1.036	1.021-1.051	<0.001	1.019	1.001-1.037	0.041
NLR									
<3.65	1	–	–	1	–	–	1	–	–
≥3.65	2.114	1.459-3.064	<0.001	2.122	1.462-3.079	<0.001	1.146	0.750-1.752	0.528
PLR (1sd)	1.001	1.000-1.002	0.004	1.001	1.000-1.002	0.017	1.000	1.000-1.001	0.286
PLR									
<130.95	1	–	–	1	–	–	1	–	–
≥130.95	1.872	1.294-2.709	0.001	1.878	1.291-2.732	0.001	1.309	0.869-1.972	0.198
MHR (1sd)									
MHR	1.092	0.661-1.804	0.731	1.126	0.682-1.862	0.642	0.881	0.507-1.532	0.655
<0.41	1	–	–	1	–	–	1	–	–
≥0.41	0.744	0.516-1.071	0.112	0.744	0.533-1.124	0.179	0.765	0.430-1.035	0.071

NLR, neutrophil-lymphocyte ratio; PLR, platelet–lymphocyte ratio; MHR, lymphocyte-monocyte ratio; OR, odds ratio; CI, confidence interval.

Model 1: adjusted for age, sex, smoking.

Model 2: adjusted for age, sex, smoking, PaO2/FiO2, white blood cell count, lactate dehydrogenase, total cholesterol, apolipoprotein B, carcinoembryonic antigen, cytokeratin 21-1 and neurospecific enolase.

### NLR as a Predictor for Overall Survival in All Patients

**Figure 5:**
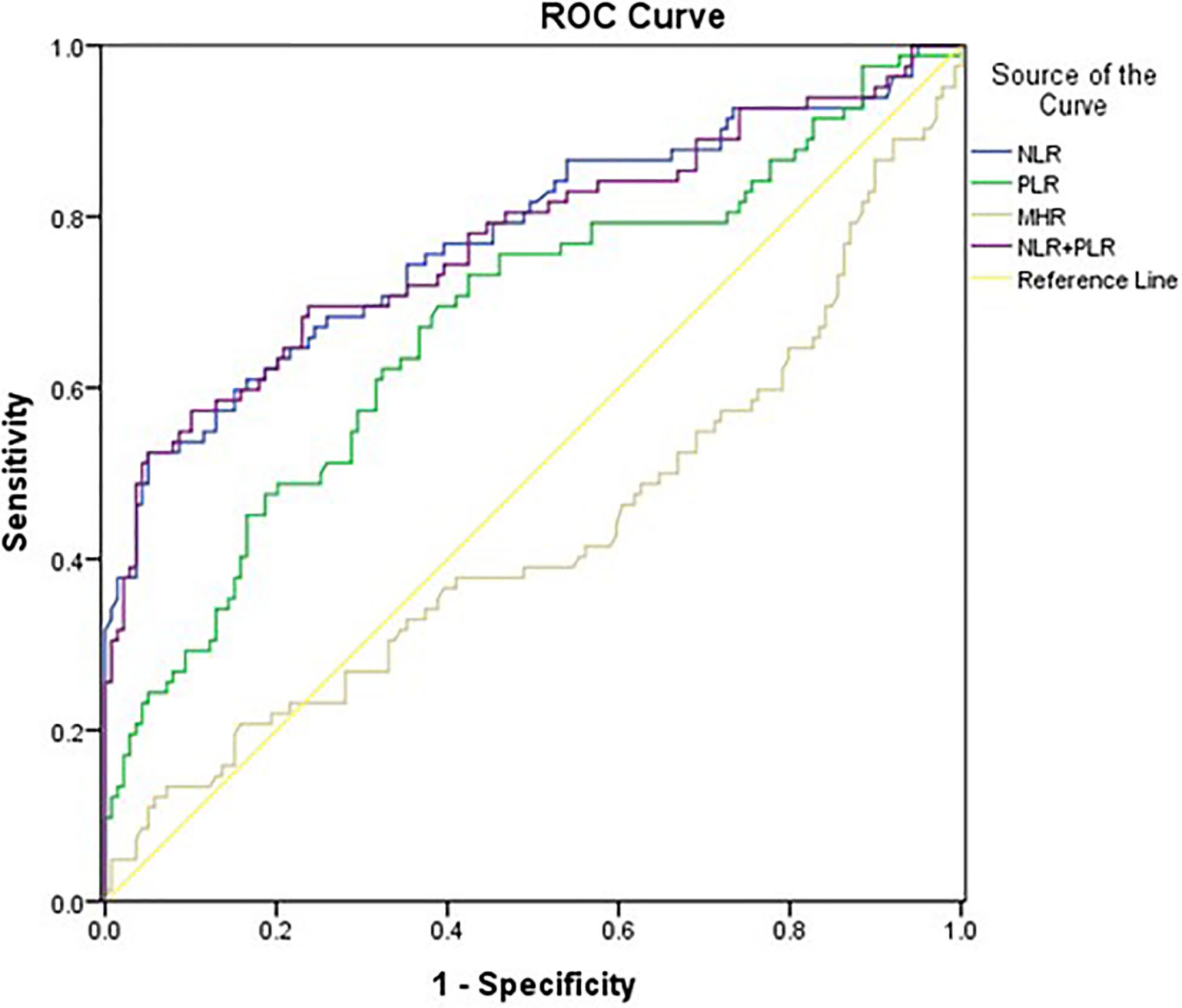
Area under the receiver operating characteristics curve (AUROC) of the NLR, PLR and MHR for overall survival.

The AUROC of NLR (0.776, 95% *CI* 0.707-0.844, *P*<0.001) was larger than that of PLR (0.676, 95% *CI* 0.600-0.751, *P*<0.001) and MHR (0.430, 95% *CI* 0.348-0.512, *P*=0.082). In addition, we combined NLR and PLR to evaluate the prognosis value for overall survival. The AUROC of NLR+PLR was (0.772, 95% *CI* 0.703-0.842, *P*<0.001) (**Figure 5**).

### Association Between Baseline NLR/PLR/MHR and Overall Survival in AE-IPF Patients

The baseline characteristics of the AE-IPF subgroup are listed in **Supplementary Table 1**. After adjusted for confounding factors including age, sex, PaO2/FiO2, WBCs, LDH, apoB, D-dimer, CEA, CYFRA21-1 and NSE, NLR was an independent risk factor for overall survival (*OR*=1.022, 95% *CI* 1.001-1.044, *P*=0.036) in patients with AE-IPF. However, PLR *(OR*=1.001, 95% *CI* 0.999-1.002, *P*=0.298) and MHR(OR= 0.977, 95% *CI* 0.453-2.107, *P*=0.953) were not significantly correlated with overall survival in patients with AE-IPF, after adjusted for confounding factors.

### Changes in NLR/PLR/MHR on Last Admission and Before Death in AE-IPF Patients Who Experienced In-Hospital Death

The baseline characteristics of the last admission in 34 AE-IPF patients who underwent in-hospital mortality are summarized in **Supplementary Table 2**. In view of the significant differences between last admission and before death, we performed a Mann–Whitney analysis. The NLR before death was significantly higher than that on admission (21.52 ± 13.48 *vs* 15.17 ± 16.96, *P*=0.014). There were no significant changes in PLR and MHR on admission or before death (PLR: 263.18 ± 188.25 *vs* 274.15 ± 325.59, *P*=0.739; MHR: 0.52 ± 0.42 *vs* 0.53 ± 0.60, *P*=0.478). There was no significant correlation between the three prognostic factors on admission and the length of hospitalization.

## Discussion

IPF is a chronic progressive lung disease characterized by parenchymal fibrosis of unknown origin leading to irreversible lung injury (21). The incidence of IPF is estimated to range from 1.25 to 23.4 cases per 100,000 population. The incidence of IPF is specifically high in male adults aged over 50 years. The 5-year survival rate of IPF patients is approximately 20% (6). It is acknowledged that accurate assessment of the severity of IPF disease is critical to determine therapeutic strategies. Thus, it is necessary to find simple, inexpensive and easily accessible indicators to identify severe cases of IPF, which will be useful to provide personalized treatment in advance and improve survival rates.

In this retrospective cohort study, we confirmed that NLR and PLR were significantly negatively correlated with PaO2/FiO2. There was a significant negative correlation between baseline NLR and overall survival of patients with IPF. A higher NLR on admission was associated with a shorter overall survival time in both the total cohort and the AE-IPF subgroup. The relationship remained stable after adjusting for clinical confounders. Moreover, we found that in AE-IPF patients who experienced in-hospital mortality, the NLR dynamically increased during the last hospital stay. ROC analysis showed that the AUROC of NLR for predicting overall survival was 0.776, larger than that of PLR, MHR and NLR+PLR. These results indicated that NLR might serve as a potential prognostic biomarker for overall survival in patients with IPF.

To our knowledge, there have been only four previous studies on the relationship between baseline NLR or PLR and the clinical outcomes of IPF patients. Poor prognosis is observed in patients with a higher baseline NLR and is independent of the GAP (sex, age, physiology) score. A previous retrospective study of 73 IPF patients indicated that the NLR was significantly negatively associated with baseline forced expiratory volume in the 1st second (FEV1) and diffusion capacity for carbon monoxide (DLCO%) (16). Nathan et al. found that a higher increase in NLR or PLR over 12 months was positively associated with higher mortality (15). D’alessandro et al. measured the NLR in bronchoalveolar lavage (BAL) samples isolated from IPF patients, indicating a negative correlation between NLR and forced vital capacity (FVC) and FEV1 (17). Their results indicated that NLR was correlated with the composite physiologic index, which was measured at the timepoint of collecting BAL samples. The relationship between baseline NLR and prognosis in IPF patients was consistent with previous studies. To the best of our knowledge, this is the first time that the NLR, PLR and MHR have been monitored to identify their association with IPF outcomes.

Although the mechanism of IPF has not been fully elucidated, the aging of the alveolar epithelium is remarkably associated with the progression of pulmonary fibrosis (6). Recurrent epithelial injury is triggered by comprehensive genetic and environmental risk factors, such as shortened telomeres, oxidative injury, proteostatic dysregulation, and endoplasmic reticulum stress, followed by uncontrolled migration and proliferation of lung fibroblasts and differentiation from fibroblasts to myofibroblasts. Pulmonary fibrosis is the end result of chronic inflammation, aberrant wound repair and acceleration, which leads to irreversible damage to lung tissue (22, 23). Collectively, inflammation and oxidative stress play a central role in the fibrogenesis of lung fibrosis.

Transforming growth factor-β (TGF-β)-mediated signaling is one of the critical pathways in the excessive inflammatory response and is required for the development of IPF (24). Neutrophil elastase (NE) is a serine proteinase generated by active neutrophils that is highly similar to components of the extracellular matrix. Several studies indicate that NE enhances pulmonary fibrosis by inducing fibroblast proliferation and myofibroblast differentiation in a TGF-β-independent pathway (25, 26). Monocytes also promote fibrosis formation through two critical mechanisms: one is to produce proinflammatory cytokines [IFN-α, MIB-α (CCL3) and MIP-1β (CCL4)] to promote myofibroblast differentiation, and the other way is to serve as progenitor fibrocytes to induce pulmonary fibrosis (27). Platelets can also participate in fibrogenesis by modulating the production of TGF-β1 by platelet-derived growth factor. Recurrent alveolar epithelium injuries can activate platelets with fibrin-rich clot formation, resulting in upregulation of plasminogen activator inhibitor-1 (PAI-1) by inducing the activation of TGF-β1/Smad3 signaling (24). High-density lipoprotein cholesterol (HDL-c) plays an anti-inflammatory and antioxidant role through the expression of endothelial nitric oxide synthase to decrease the vascular tension of endothelial cells and accelerate the oxidation of low-density lipoprotein cholesterol (LDL-c) (28).

It is well known that pulmonary function tests, such as percent predicted FVC and percent predicted DLCO, are used to evaluate the severity of lung impairment and predict prognosis in IPF patients (20). In our study, approximately half of the patients experienced respiratory failure and were not able to cooperate with lung function tests. Instead, because the PaO2/FiO2 test has become routine in the clinical diagnosis and treatment of IPF, we chose PaO2/FiO2 to reflect lung function because PaO2/FiO2 decline is among the earliest objective indicators of abnormal gas exchange function, and PaO2/FiO2 measurement is a routine test in our center. We found that the expression of NLR and PLR was negatively correlated with PaO2/FiO2. In addition, in the subgroup of AE-IPF patients, there was a dynamic increase in NLR at the last measurement compared with that at the last admission, which indicates the potential prognostic value of early changes in these inflammatory biomarkers. Because complete repetitive measurement data of these inflammatory biomarkers are still limited, large, randomized, multicenter, prospective studies are needed to verify our hypothesis.

Our results should be interpreted within the limitations of the study. First, this was a small-sample retrospective single-center study. Second, we did not evaluate the lung function of these participants because lung function test was difficult for the severer patients. Finally, there were no regular repetitive measurements of these prognostic biomarkers at certain points, so we could not sufficiently evaluate the changes in these parameters as disease progressed.

In conclusion, in our study, there was a negative correlation between baseline NLR and PaO2/FiO2. NLR was an independent prognostic factor of shorter overall survival time in the total cohort of IPF patients and in AE-IPF patients. Furthermore, we found that there was a significant increase in NLR at the last measurement compared with that at the last admission in nonsurvival AE-IPF patients. We should pay more attention to NLR expression and identify patients with poor outcomes in advance to obtain a better prognosis. However, prospective studies are needed to prove our findings.

## Data Availability Statement

The original contributions presented in the study are included in the article/**Supplementary Material**. Further inquiries can be directed to the corresponding author.

## Ethics Statement

The studies involving human participants were reviewed and approved by Nanjing Drum Tower Hospital ethics committee. The patients/participants provided their written informed consent to participate in this study.

## Author Contributions

XY and YC conceived and designed the study. JC and MZ collected the data. XY, YC, JC, and MZ analyzed the data. YC wrote the original draft. XY, JC, and MZ revised the manuscript. XY administered and supervised the study. All authors contributed to the article and approved the submitted version.

## Conflict of Interest

The authors declare that the research was conducted in the absence of any commercial or financial relationships that could be construed as a potential conflict of interest.

The reviewer CW declared a shared parent affiliation with the authors to the handling editor at the time of the review.

## Publisher’s Note

All claims expressed in this article are solely those of the authors and do not necessarily represent those of their affiliated organizations, or those of the publisher, the editors and the reviewers. Any product that may be evaluated in this article, or claim that may be made by its manufacturer, is not guaranteed or endorsed by the publisher.
