# Supplementary material for: Prognostic Role of NLR, PLR and MHR in Patients With Idiopathic Pulmonary Fibrosis
Source: Front Immunol. 2022 Apr 28;13:882217. doi: 10.3389/fimmu.2022.882217 (PMC9096781; doi:10.3389/fimmu.2022.882217)
Supplement: Supplementary file 1 [file Table_1.docx]

**Prognostic role of NLR, PLR and MHR in patients with** **idiopathic pulmonary fibrosis**

Yiran Chen^1^,Jingya Cai^2^,Mengmeng Zhang^2^,Xin Yan^2*^

^1^Medical school of southeast university Nanjing Drum Tower Hospital, Nanjing 210000, China

^2^Department of respiratory and critical care medicine, Drum Tower Hospital, Nanjing University School of Medicine, Nanjing 210000, China

Corresponding author: Xin Yan. Address: Department of respiratory and critical care medicine, Drum Tower Hospital, Nanjing University School of Medicine, Nanjing 210000, China; <Tel:15365117831>; e-mail: [yanxin8612@126.com](mailto:yanxin8612@126.com); ORCID: yanxin8612@126.com

| **Supplement Table 1** Comparison of baseline characteristics between AE-IPF and stable IPF patients | | | |
| --- | --- | --- | --- |
| Variables | AE | Stable | *P* |
|  | (n=116) | (n=162) |  |
| Age(years) | 68.68±8.41 | 67.46±9.28 | 0.409 |
| Male(n, %) | 92(79.31) | 128(79.01) | 0.952 |
| Hypertension(n, %) | 46(39.66) | 47(29.01) | 0.064 |
| Diabetes(n, %) | 25(21.55) | 36(22.22) | 0.894 |
| Rheumatic diseases(n, %) | 18(15.52) | 33(20.37) | 0.303 |
| Smoking(n, %) | 49(42.24) | 58(36.42) | 0.326 |
| PAO2/FiO2 | 179.70±68.32 | 389.54±79.93 | 0.000 |
| WBC count(10^9/L) | 9.29±4.10 | 7.39±2.54 | 0.000 |
| Neutrophils (10^9/L) | 7.18±3.87 | 4.96±2.34 | 0.000 |
| Lymphocytes(10^9/L) | 1.46±0.81 | 1.71±0.69 | 0.002 |
| Monocytes(10^9/L) | 0.48±0.33 | 0.49±0.21 | 0.109 |
| Platelet(10^9/L) | 211.99±95.34 | 193.35±75.32 | 0.197 |
| ALT(U/L) | 22.90±16.57 | 21.37±16.31 | 0.472 |
| AST(U/L) | 22.52±12.09 | 20.00±8.42 | 0.166 |
| LDH(U/L) | 361.37±170.08 | 245.02±65.56 | 0.000 |
| ALP(U/L) | 79.97±27.83 | 71.29±21.25 | 0.003 |
| TB (umol/L) | 11.34±5.89 | 10.36±4.76 | 0.356 |
| TC(mmol/L) | 4.21±1.05 | 4.23±1.00 | 0.537 |
| HDL(mmol/L) | 1.04±0.35 | 1.16±0.36 | 0.002 |
| LDL(mmol/L) | 2.40±0.78 | 2.49±0.76 | 0.388 |
| ApoA(g/L) | 0.90±0.26 | 1.04±0.27 | 0.000 |
| ApoB(g/L) | 0.82±0.26 | 0.78±0.21 | 0.427 |
| CRP(ng/L) | 37.54±44.43 | 12.33±21.86 | 0.000 |
| D-dimer（mg/L) | 4.54±14.68 | 1.00±2.16 | 0.000 |
| CEA（ng/ml) | 5.39±4.08 | 3.37±2.46 | 0.000 |
| CYFRA21-1(ng/ml) | 7.97±4.77 | 4.54±2.21 | 0.000 |
| NSE(ng/ml) | 20.43±9.19 | 14.84±5.02 | 0.000 |
| NLR | 8.36±11.45 | 4.38±4.98 | 0.000 |
| PLR | 206.07±213.59 | 148.21±100.17 | 0.003 |
| MHR | 0.53±0.48 | 0.50±0.33 | 0.404 |

**Abbreviations**: AE: acute exacerbation; WBC: white blood cell; ALT: alanine aminotransferase; AST: glutamic oxaloacetic transaminase; LDH: lactate dehydrogenase; ALP: alkaline phosphatase; TC: total cholesterol; TB：total biliburin; HDL: high density lipoprotein; LDL: low density lipoprotein; ApoA: Apolipoprotein A; ApoB: Apolipoprotein B; CRP: C-reactive protein; CEA: carcinoembryonic antigen; CYFRA21-1: cytokeratin 21-1; NSE: neurospecific enolase; NLR: the neutrophil-lymphocyte ratio; PLR: the platelet–lymphocyte ratio; MHR: the monocyte-high density lipoprotein ratio.
