# Supplementary material for: Prognostic Role of NLR, PLR and MHR in Patients With Idiopathic Pulmonary Fibrosis
Source: Front Immunol. 2022 Apr 28;13:882217. doi: 10.3389/fimmu.2022.882217 (PMC9096781; doi:10.3389/fimmu.2022.882217)
Supplement: Supplementary file 2 [file Table_2.docx]

**Prognostic role of NLR, PLR and MHR in patients with** **idiopathic pulmonary fibrosis**

Yiran Chen^1^,Jingya Cai^2^,Mengmeng Zhang^2^,Xin Yan^2*^

^1^Medical school of southeast university Nanjing Drum Tower Hospital, Nanjing 210000, China

^2^Department of respiratory and critical care medicine, Drum Tower Hospital, Nanjing University School of Medicine, Nanjing 210000, China

Corresponding author: Xin Yan. Address: Department of respiratory and critical care medicine, Drum Tower Hospital, Nanjing University School of Medicine, Nanjing 210000, China; <Tel:15365117831>; e-mail: [yanxin8612@126.com](mailto:yanxin8612@126.com); ORCID: yanxin8612@126.com

**Supplement Table 2** Baseline characteristics of 34 AE-IPF patients experienced in-hospital mortality

| Variables | AE-IPF patients experienced in-hospital mortality |
| --- | --- |
|  | (n=34) |
| Age(years) | 70.03±8.77 |
| Male | 25(73.53%) |
| Hypertension | 10(29.41%) |
| Diabetes | 5(14.71%) |
| Rheumatic diseases | 6(17.65%) |
| Smoking, n% | 11(32.35%) |
| PAO2/FiO2 | 181.74±86.52 |
| WBC count(10^9/L) | 10.47±4.85 |
| Neutrophils (10^9/L) | 8.32±4.62 |
| Lymphocytes(10^9/L) | 1.46±0.95 |
| Monocytes(10^9/L) | 0.50±0.39 |
| Platelet(10^9/L) | 210.21±109.92 |
| ALT(U/L) | 22.08±11.09 |
| AST(U/L) | 23.66±12.49 |
| LDH(U/L) | 461.74±223.28 |
| ALP(U/L) | 85.74±38.67 |
| TB (umol/L) | 11.80±6.85 |
| TC(mmol/L) | 4.63±0.97 |
| HDL(mmol/L) | 1.15±0.33 |
| LDL(mmol/L) | 2.73±0.91 |
| ApoA(g/L) | 0.99±0.30 |
| ApoB(g/L) | 0.92±0.30 |
| CRP(ng/L) | 36.66±56.93 |
| D-dimer（mg/L) | 7.39±20.16 |
| CEA（ng/ml) | 6.99±4.94 |
| CYFRA21-1(ng/ml) | 9.79±5.74 |
| NSE(ng/ml) | 20.98±8.14 |
| NLR | 11.76±16.32 |
| PLR | 246.12±325.66 |
| MHR | 0.50±0.57 |

**Abbreviations**: AE: acute exacerbation; WBC: white blood cell; ALT: alanine aminotransferase; AST: glutamic oxaloacetic transaminase; LDH: lactate dehydrogenase; ALP: alkaline phosphatase; TC: total cholesterol; TB：total biliburin; HDL: high density lipoprotein; LDL: low density lipoprotein; ApoA: apolipoprotein A; ApoB: apolipoprotein B; CRP: C-reactive protein; CEA: carcinoembryonic antigen; CYFRA21-1: cytokeratin 21-1; NSE: neurospecific enolase; NLR: the neutrophil-lymphocyte ratio; PLR: the platelet–lymphocyte ratio; NLR: the neutrophil-lymphocyte ratio; PLR: the platelet–lymphocyte ratio; MHR: the monocyte-high density lipoprotein ratio.
